# Innovative Demand Creation for Voluntary Medical Male Circumcision Targeting a High Impact Male Population: A Pilot Study Engaging Pregnant Women at Antenatal Clinics in Kampala, Uganda

**DOI:** 10.1097/QAI.0000000000001041

**Published:** 2016-10-06

**Authors:** Aggrey S. Semeere, Barbara Castelnuovo, Denis S. Bbaale, Agnes N. Kiragga, Joanita Kigozi, Alex M. Muganzi, Alex G. Coutinho, Andrew Kambugu

**Affiliations:** Research and Outreach Departments, Infectious Diseases Institute, Makerere University College of Health Sciences, Kampala, Uganda.

**Keywords:** voluntary medical male circumcision, pregnant women

## Abstract

Circumcision has been shown to be an effective method of HIV prevention; however, only 28% of Ugandan men aged 15–49 years are circumcised. There is a paucity of data on the role of intimate partners in generating demand for voluntary medical male circumcision (VMMC). We conducted a pilot study to assess the feasibility of a partner-focused intervention targeting males >25 years.

**Methods:**

Among pregnant women in their third trimester attending antenatal care we evaluated the impact of a pilot behavior change intervention on VMMC through a quasi-experimental approach. We observed VMMC numbers among spouses of women as per standard practice (comparison phase), and after introducing a behavioral change communication package (intervention phase). Logistic regression was used to compare the odds of VMMC uptake between comparison and intervention phases. We used qualitative methods to evaluate the casual chain using a thematic approach.

**Results:**

Of the 601 women studied, 90% articulated the health benefits of VMMC and 99% expressed interest in their spouse getting circumcised. Women's knowledge was not increased by the intervention. Four men were circumcised in the comparison and 7 in the intervention phase. The intervention was not associated with higher odds of circumcision (odds ratio 1.5, 95% CI: 0.3 to 6.0, *P* = 0.65). We interviewed 117 individuals overall with the main enablers for VMMC being: free VMMC, transport reimbursement, and health benefits. Deterrents included misconceptions, lost wages and fear of pain. Most of the uncircumcised men interviewed reported interest in VMMC.

**Conclusions:**

Our pilot intervention had no significant impact on increasing VMMC demand. The study demonstrated the feasibility of pregnant women engaging their spouses to discuss VMMC.

## INTRODUCTION

Landmark clinical-trials^1–3^ and observational studies^4–6^ have demonstrated that circumcision significantly reduces HIV acquisition risk among heterosexual men. By 2013, only 28% of Ugandan men aged 15–49 years are circumcised^7^ and prevalence of HIV infection was 7.3% in 2011.^8^ Initiatives to increase the proportion of circumcised males have been tried such as task-shifting^3,9^ and use of nonsurgical approaches^10,11^ but all are yet to substantially increase this proportion. The Infectious Diseases Institute (IDI) offers voluntary medical male circumcision (VMMC) services in the Ugandan capital city (Kampala).^12^ Despite initial success, monthly circumcision numbers (2800–3500) are below the 4500 target.^13^ A survey of communities served by IDI revealed that the demand-generation activities were too general to address the individual barriers of men.^13^ Key individual barriers included fear (of pain, adverse outcomes, and lost wages), misinformation, access to services, and stand-alone services.

There is a need to explore strategies to escalate demand, including engaging females partners,^14–17^ but little evidence exists of how this can be implemented.^18^ Women are considered the health custodians in communities and are a possible source of demand generation for circumcision, given their influence on men by providing the right information and guidance.^19^ Pregnancy offers the unique opportunity to align puerperium with the 6–8 week period of abstinence after circumcision which is essential for healing. This period of abstinence has been noted as a challenge for both men and women.^20^ Alignment provides the possibility of mutual support. In this pilot study, we evaluated an approach to increase the uptake of circumcision within the context of an integrated antenatal care setting and a circumcision project in Uganda.

## METHODS

Using a quasi-experimental study design, we evaluated an intervention to increase uptake of VMMC among male partners of women in the third trimester of pregnancy receiving antenatal services at 3 high-volume Kampala clinics between May 2014 and January 2015. We compared the intervention group to a preintervention (historical) group.

The 3 study clinics, Kisugu, Kisenyi, and Kawaala health centers have been supported by IDI since 2008 and are within 5 km of each other. VMMC is performed at the Kisenyi site. All have well-attended, free antenatal services. The standard of care with respect to VMMC demand generation in these clinics consisted of occasional messaging in the patient waiting area on the availability of VMMC.

We recruited pregnant women 18 years and over with uncircumcised male partners and who had completed 30 weeks of gestation and attended the study clinics. We excluded women in labor, those not living with their intimate partner, and women with history of domestic violence with current partner. We hypothesized that a power of 80%, with an uptake of 6%, and an increase of 8% in VMMC would require a sample size of 478 women, which was increased to 600 to account for losses.

### Intervention Design and Measurements

**FIGURE 1.**
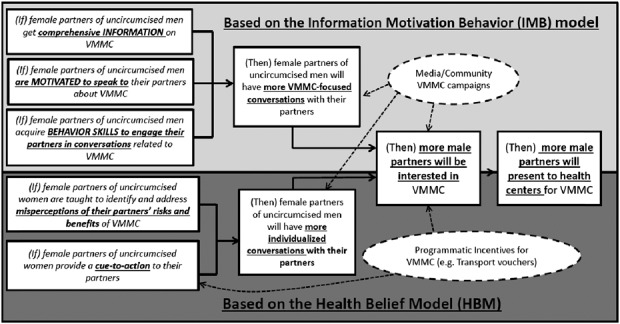
Theory of change diagram.

We applied 2 theoretical models (the information, motivation, and behavioral skill model^21–23^ and the health belief model^24,25^) to inform the theory of change for the study (Fig. 1). We used the socio-ecologic model to envisage potential influences on the key study outcomes that are not related to the intervention,^26,27^ (Fig. 1).

**FIGURE 2.**
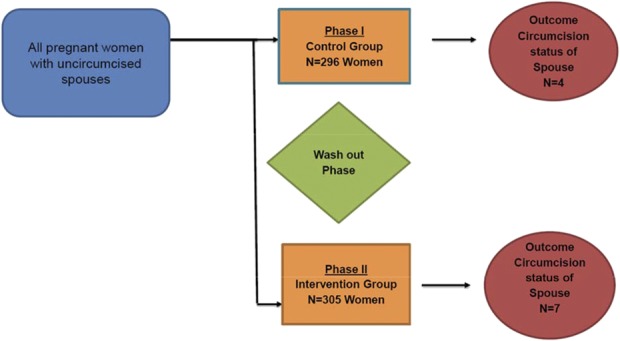
Quasi-experimental study design.

This evaluation (Fig. 2) was performed in 3 phases; (1) comparison (historical) phase (May–August 2014), (2) washout (August and September 2014), and (3) intervention phase (October and December 2014). During the comparison phase, no intervention was tested and women received standard of care and information about an $8.50 transport voucher for the male partner presenting for VMMC services. During this phase, we collected data used to develop the intervention and piloted the intervention tools in a nonstudy but similar site. Information was collected from women through a questionnaire and also from the spouses who subsequently sought VMMC. This was followed by the washout phase where no information was collected. This period was used to prevent potential contamination in case women visited the health facilities more than once. The intervention involved educating the women about the benefits of VMMC to both them and their spouse, details regarding the procedure, wound care to enhance healing, possible complications (and what to do if any occurred), as well as how and when to get the procedure performed. They were also given communication skills regarding how and when to talk to their spouse, as well as role playing to enable them to deliver the message to the spouse. The women were given a transport voucher for the spouse and an information brochure. Men were allowed up to 1 month to return for circumcision in both comparison and intervention phases of the study.

Using semistructured interviews, we collected qualitative information from both pregnant women and their spouses in both comparison and intervention phases. For those women whose spouse did not have circumcision, 10% were recruited in each of the phases for telephone interviews. We interviewed spouses of these sampled women after the women provided verbal consent and a telephone contact for their spouse. The man provided verbal consent before the interview. The interviews with the women focused on communication with the spouse and his response. Men's interviews focused on understanding their response (undergoing VMMC or otherwise) and the reasons for response to the spouse's message. For provider views regarding the intervention within an antenatal clinic setting, we interviewed key informants, including midwives, registered nurses, and counselors in the clinics. To ascertain whether spouses accessed VMMC services at the designated site, the site circumcision logs were comprehensively reviewed by the study team, including the documentation for study-coded transport vouchers.

### Statistical Analysis

We compared demographic characteristics using χ^2^ and Wilcoxon rank-sum tests. To understand the level of VMMC knowledge in both phases, we generated a knowledge score using binary responses to 7 questions asked to the women presented as a percent score. The highest score was 100 and the lowest was 0. We used a mixed-methods approach to analyze the main outcome of circumcision within 1 month of interaction with the spouse, irrespective of the site.

Impact of the intervention on circumcision was a quantitative analysis using logistic regression to compare the odds of being circumcised between the intervention and comparison groups. We adjusted for confounding from the site of recruitment, age of the woman, weeks of amenorrhea, level of education, occupation, and measures of socio-economic status. We evaluated for heterogeneous effects using multiplicative interaction. We used a *P*-value cutoff of <0.1 for statistical significance of heterogeneity. All quantitative analyses were performed using STATA 13.1.

The manifest approach of content analysis was used to understand the causal chain in the theory of change.^28^ An analysis plan based on the study objectives was used to generate a coding scheme after reading the interview transcripts. Responses that seemed to speak to a specific trend were grouped together to understand patterns in the data. This resulted in overarching themes, which provided insight to participants' perceptions of the benefits of VMMC and how information on VMMC may have impacted decisions to seek VMMC. We did not use electronic software to analyze the qualitative data.

The research protocol was approved by the Joint Clinical Research Center Institutional Review Board and regulatory approval was obtained from the Uganda National Council of Science and Technology, registration number SS-3418. This evaluation was registered by The Registry of International Development Impact Evaluations.

## RESULTS

Overall, 601 women and 11 men (Fig. 2) participated. Generally, demographic characteristics of the women in the comparison and intervention phase were similar (Table 1). Most of the women were recruited from Kisenyi (52%) and others from Kawaala (39%) and Kisugu (9%) clinics. Overall, median age of the women was 24 (interquartile range (IQR); 21–29) years, with a median of 34 (IQR; 32–37) weeks of amenorrhea. Eighty-four percent reported being in monogamous relationships. Regarding social economic status, most of the women reported a household income <500,000 shillings ($167) per month and lived in rented homes. Median rental expenditure was 70,000 shillings ($24) per month. Almost 90% of the women had at least primary or secondary education but with 50% formally unemployed (Table 1). The median age for the spouses was 29.5 (IQR; 28–34) years, 90% self-identified as being in a monogamous relationship and living with their spouse and reported a median of 1 sexual partner in the past 6 months.

**TABLE 1.**
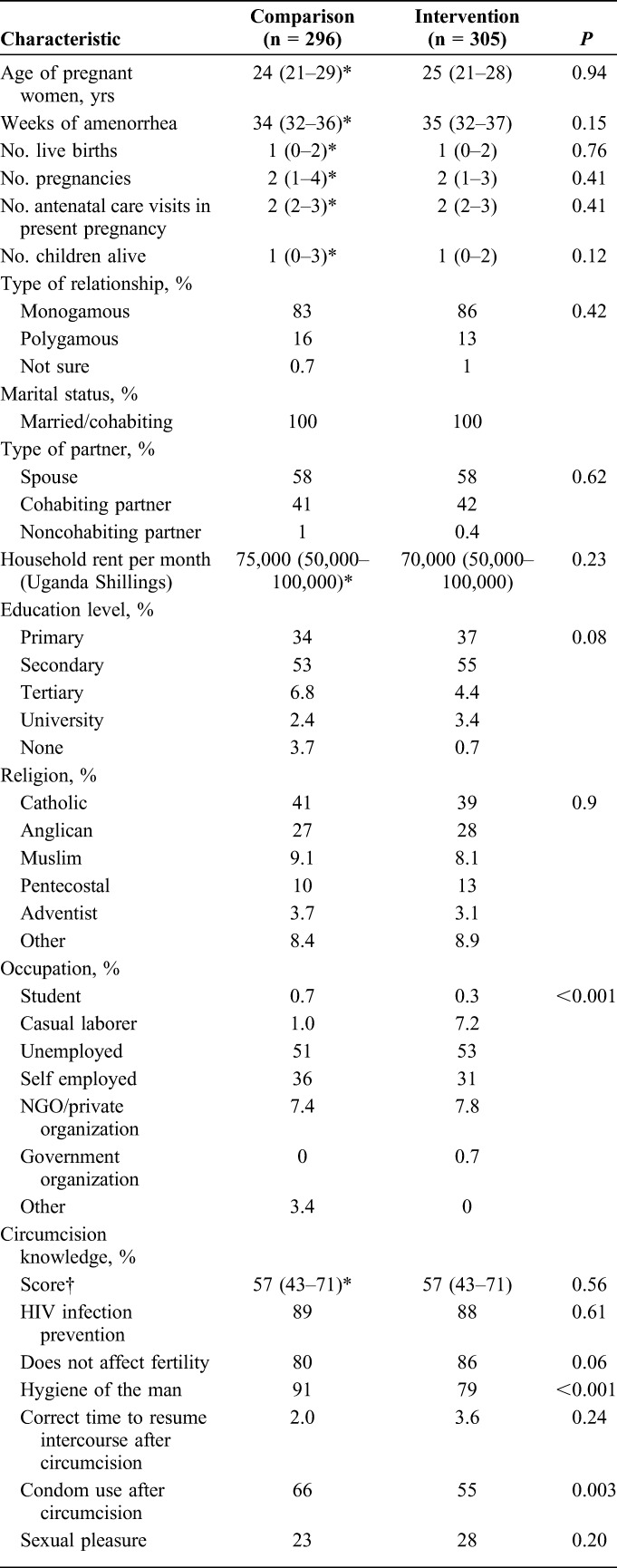

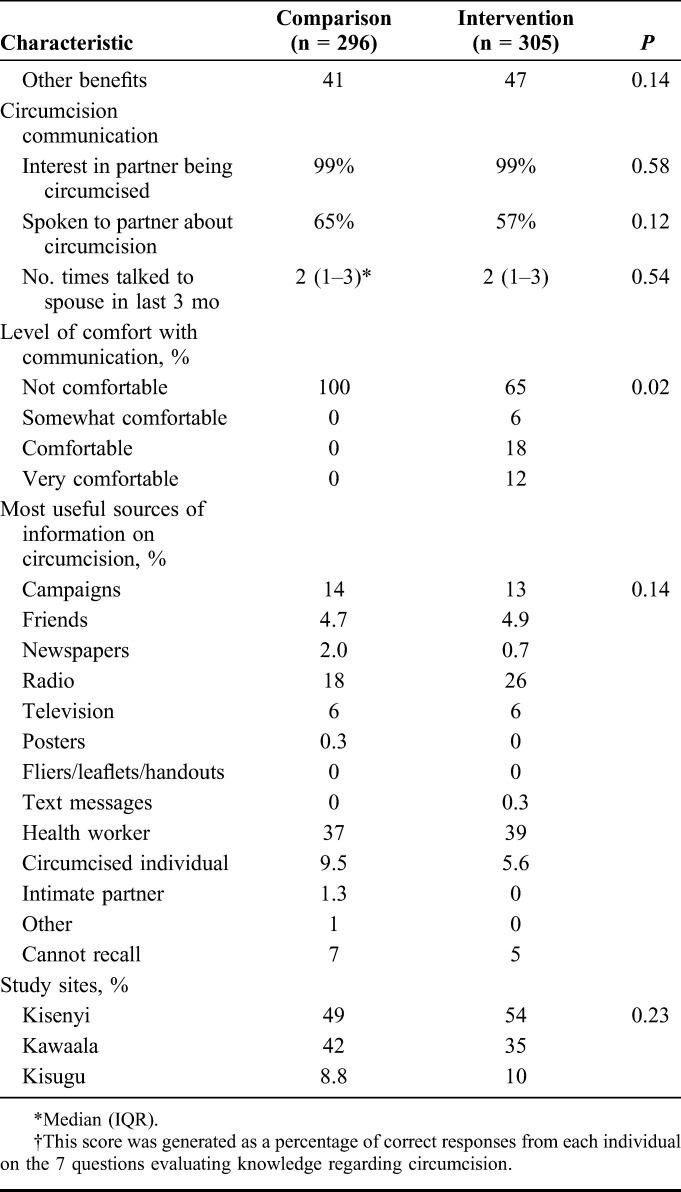
Characteristics of the Women Who Participated in the Evaluation Comparing the Comparison and Intervention Phase Populations

Before the intervention, almost 90% of the women understood the benefits of circumcision with their most important information source being a village campaign, health worker, media (radio, television), or a friend or circumcised individual (Table 1). Not clear to them was when to resume sexual intercourse after circumcision and other benefits like Human Papilloma Virus infection prevention. Knowledge scores were not higher in women in the intervention phase. Compared with women in Kisugu, women in Kisenyi and Kawaala had higher mean knowledge scores in both comparison and intervention phases with the highest difference in the intervention phase (*P* < 0.001).

Of the women, (99%) reported communicating with their spouse about circumcision. In fact, 61% (183) of the women in the comparison phase reported attempts to discuss circumcision with their spouse but also reported that they were not comfortable with the subject (Table 1).

Overall, 11 men were circumcised. Mean time between interacting with the women to returning for VMMC was 30.2 days. Median age of the men was 29.5 (IQR; 28–34) years. Four men were circumcised in the in the comparison phase and 7 in the intervention phase (odds ratio 1.5, 95% CI: 0.4 to 5.2, *P* = 0.56). Adjusting for study site, age, weeks of amenorrhea of the spouse, education level, occupation, and household income, the intervention was not associated with a higher likelihood of circumcision (odds ratio: 1.4, 95% CI: 0.3 to 6.0, *P* = 0.65). Being at >36 weeks of amenorrhea was the only factor associated with higher odds of circumcision (3.4, 95% CI: 0.8 to 14, *P* = 0.09). Other factors, including household income, occupation, marital status, study site, partner type, knowledge score, and religion were not associated with higher circumcision rates.

We interviewed 117 participants, including 32 women and 14 men in the comparison phase, and 34 women and 16 men in the intervention phase, and 21 key informants (Table 2).

**TABLE 2.**
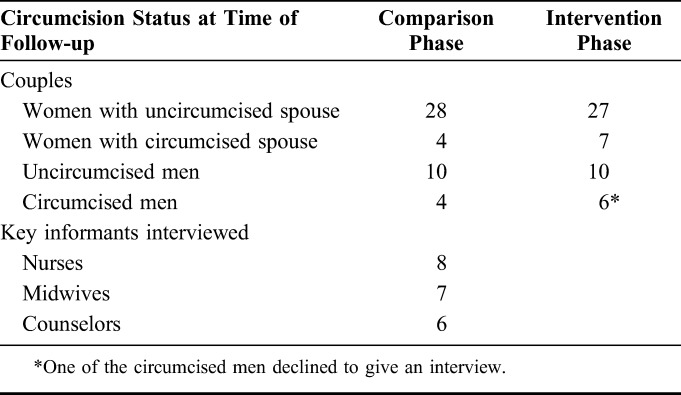
Summary of the Breakdown of Interviewed Participants for the Qualitative Data Collection Including Circumcision Status of the Couples During the Follow-up

### Empowerment of Women With Information and Communication Skills

Most interviewed women agreed that the intervention imparted knowledge regarding circumcision benefits and procedures and emboldened them to engage with their partners. The intervention provided comprehensive understanding, made them more aware of the barriers to VMMC and how they can be overcome.

Almost all the women (99%) in the intervention phase reported delivering the message to their partners, and this was confirmed by the interviewed male partners. The women shared positive and negative experiences and most stated that their spouses were willing to be circumcised after their encouragement. The following quote serves to illustrate this:

He allowed and promised to go the week after …. he wanted me to first give birth and in case the baby is a boy, they take him to also get circumcised.

The negative responses had social-cultural intonations as evidenced below.

I told him but his family had just cautioned him not to circumcise. He told me to just shut up, …. he knows about circumcision.

For those whose spouses did not turn up for circumcision, women attributed this to barriers such as religious and cultural perceptions, fear of pain, lack of time, being older, and hence VMMC irrelevant. Examples include:

He also still has the thought from his culture that he shouldn't do things that his parents/elders didn't do.

…he said that Catholics are not allowed to get circumcised

He fears he may feel too much pain and yet he has to work

…he said has no time, he is busy at work and he also fears the pain

Also the time taken to heal is too long for him to ask for leave or else he would lose his job

…He cannot go 2 months without working

### Men's Response to Wives' Message

During the comparison phase, 56% of the interviewed uncircumcised men indicated willingness to get VMMC, whereas after the intervention, this proportion was 78%. All men who were circumcised in the intervention arm had interest in undergoing VMMC before getting a message from their spouses and all indicated that the discussion preempted the visit to the clinic. Emergent themes were similar in both phases in terms of the men's response to wives' message. Among those who underwent VMMC, enabling factors included desire for good hygiene and fear of contracting HIV/AIDS. The analysis indicates that these men already had some interest and that the decision was triggered by the discussion as illustrated below:

…I was already interested so it was just the right time for her to bring it up. …it brought more emphasis for me to go for the circumcision.

I was already interested in getting circumcised because it's good and has a number of advantages to men.

This made me become interested in the circumcision and eventually go for it.

For male partners who did not present for VMMC especially during the comparison phase, emerging barriers to VMMC were related to stigma associated with being seen in a “skirt” (since one cannot wear pants after the procedure) and fear of being perceived as promiscuous. A minority revealed inadequate information like the duration of the healing process and resources (not knowing VMMC services were free) as being inhibitors to uptake of VMMC. Barriers such as perceived long healing process, religious and cultural beliefs also contributed to the low uptake of VMMC. There were participants who perceived circumcision as a practice exclusive to Muslims. Some men expressed that circumcision was not part of their culture. A number of quotes are cited for emphasis:

Men from western Uganda don't circumcise.

…Circumcision is something meant for children and youths since they are the ones carelessly engaging into sex unlike me….

The interviews indicated that provision of the transport voucher greatly facilitated VMMC at the designated site:

I can go for free circumcision services at Kisenyi Health Center and be given a transport refund.

## KEY INFORMANT INTERVIEWS

From key informant interviews, we learned that the health workers had appreciable knowledge regarding the health benefits of circumcision, especially in relation to HIV prevention, but cited cultural/religious barriers to men seeking VMMC. A midwife stated that; “…*it is hard for a man to circumcise if it goes against his culture or traditional norms and beliefs.*”

They believed that women have a role in encouraging their spouses to seek VMMC and suggested that the intervention should target the couple not as individuals but as a whole with discussion with both at the same time. Despite the busy clinics, the health workers thought that with adequate training and sensitization, they can engage women to encourage their spouses come for VMMC. One registered nurse at one of the sites thought that:

“Women can easily be accessed as they come for ANC and immunization to provide them with VMMC information.”

## DISCUSSION

In this pilot study, we sought to demonstrate the potential impact of information-based partner-mediated intervention for VMMC demand generation. Despite the intervention showing a 1.5-fold increase in demand for VMMC, it was not statistically significant. A study across sub-Saharan Africa suggests that the influence of women on the acceptability and uptake of VMMC by their male counterparts is vital.^14–16,20^ However, women's influence varies across different communities based on the cultures and religious beliefs in the community.^29,30^ In this study, we sought to contribute to the body of evidence on the potential role of women on VMMC demand generation.

We explored why the intervention may not have yielded the anticipated impact on demand for VMMC. The intervention increased the level of confidence of the female partner in communicating about VMMC to their spouses (0% versus 35% in the comparison and intervention groups, respectively). It is remarkable that, whereas up to 2/3 of the participants were not comfortable with undertaking such a conversation, they nevertheless delivered the message. The high discomfort levels may have hampered effective delivery of the message and may provide understanding of why the intervention did not have the desired impact.

We did not find key differences in the delivery of the VMMC messages between the women whose partners presented for VMMC and those whose partners did not. One consistent finding was that men who presented for VMMC had contemplated undergoing VMMC and the conversation with their female partners was a tipping point for action. Most of these partners indicated that provision of a transport voucher was a catalyst to presenting at the VMMC site.

Interviews with the partners who did not present indicated key barriers previously noted, including potential lost wages; pain during recovery, and religious/cultural issues.^14,15,17,20^ Emerging literature and program experience does indicate that incentive-based interventions to overcome loss of income associated with VMMC leads to significant impact on VMMC uptake.^31^ Unfortunately, we did not directly address this issue. With respect to overcoming the barrier of pain associated with VMMC, it is possible that this specific barrier is better addressed by male peers who have undergone VMMC. Finally, the intervention did not specifically address cultural/religious barriers to VMMC. These barriers may be better addressed through the guidance of highly influential persons within communities including religious and community leaders.

### Study Limitations

This was a pilot study with time and resource constraints. The comparison and intervention period were limited to 3 months. There is a possibility that some participants presented outside this window. Furthermore, the study team only had a single interface with the female partner, and had no way of influencing any ongoing engagement of the male partner. The effect of such a modification of the intervention should be explored in further evaluations.

## CONCLUSION AND RECOMMENDATIONS

Our findings suggest that the information-based partner-mediated intervention for generating demand for VMMC is feasible and increased the level of comfort of female partners to engage their male spouses in VMMC discussions. Our recommendation is that additional studies that test a modified intervention including incentives should be explored.
